# Publisher Correction: High-throughput fluorescence lifetime imaging flow cytometry

**DOI:** 10.1038/s41467-025-55961-4

**Published:** 2025-01-10

**Authors:** Hiroshi Kanno, Kotaro Hiramatsu, Hideharu Mikami, Atsushi Nakayashiki, Shota Yamashita, Arata Nagai, Kohki Okabe, Fan Li, Fei Yin, Keita Tominaga, Omer Faruk Bicer, Ryohei Noma, Bahareh Kiani, Olga Efa, Martin Büscher, Tetsuichi Wazawa, Masahiro Sonoshita, Hirofumi Shintaku, Takeharu Nagai, Sigurd Braun, Jessica P. Houston, Sherif Rashad, Kuniyasu Niizuma, Keisuke Goda

**Affiliations:** 1 Department of Chemistry, The University of Tokyo, Tokyo, Japan; 2 Department of Neurosurgical Engineering and Translational Neuroscience, Tohoku University Graduate School of Medicine, Miyagi, Japan; 3 Department of Chemistry, Kyushu University, Fukuoka, Japan; 4 Research Institute for Electronic Science, Hokkaido University, Hokkaido, Japan; 5 Department of Neurosurgery, Tohoku University Graduate School of Medicine, Miyagi, Japan; 6 Graduate School of Pharmaceutical Sciences, The University of Tokyo, Tokyo, Japan; 7 SANKEN (The Institute of Scientific and Industrial Research), Osaka University, Osaka, Japan; 8 Miltenyi Biotec B.V. & Co. KG, Bergisch Gladbach, Germany; 9 Institute for Genetic Medicine, Hokkaido University, Hokkaido, Japan; 10 Institute for Life and Medical Sciences, Kyoto University, Kyoto, Japan; 11 Institute for Genetics, Justus-Liebig-University Giessen, Giessen, Germany; 12 Department of Chemical and Materials Engineering, New Mexico State University, Las Cruces, NM USA; 13 Department of Neurosurgical Engineering and Translational Neuroscience Graduate School of Biomedical Engineering, Tohoku University, Miyagi, Japan; 14 Institute of Technological Sciences, Wuhan University, Hubei, China; 15 Department of Bioengineering, University of California, Los Angeles, CA USA

**Keywords:** Lab-on-a-chip, High-throughput screening, Flow cytometry

Correction to: *Nature Communications* 10.1038/s41467-024-51125-y, published online 4 September 2024

The original version of this Article contained an error in Fig. 1b, in which key dashed lines were missing in the phase delay plot, and an error in Fig. 1c, where the symbol π was incorrectly denoted. The correct version of Fig. 1 is:
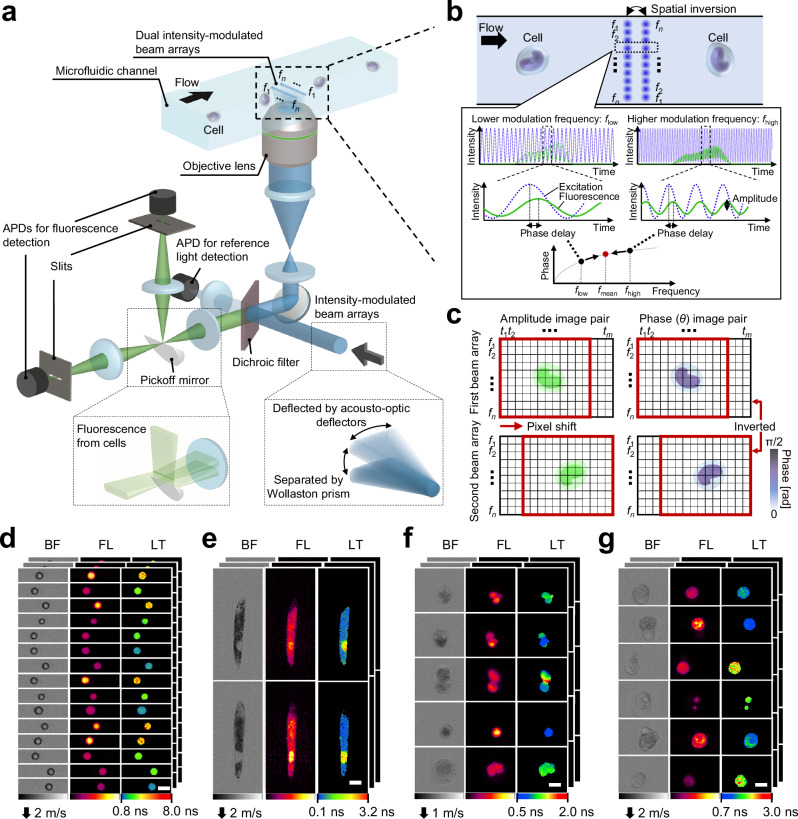


which replaces the previous incorrect version:
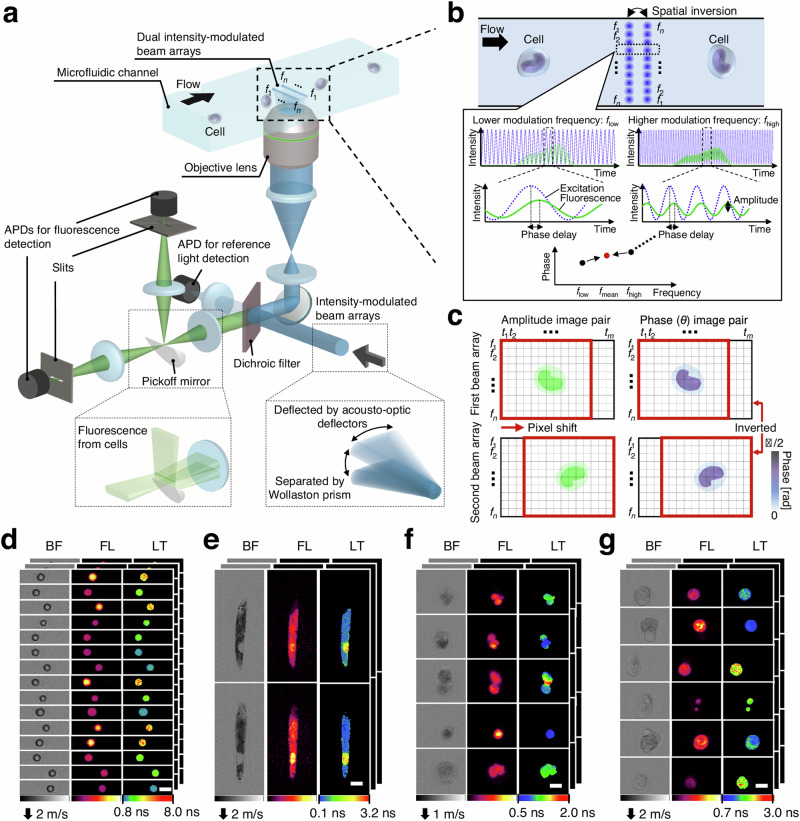


This has been corrected in both the PDF and HTML versions of the Article.

