# Supplementary material for: High-throughput fluorescence lifetime imaging flow cytometry
Source: Nat Commun. 2024 Sep 4;15:7376. doi: 10.1038/s41467-024-51125-y (PMC11375057; doi:10.1038/s41467-024-51125-y)
Supplement: Supplementary file 1 — Supplementary Information [file 41467_2024_51125_MOESM1_ESM.pdf]

High-throughput fluorescence lifetime imaging flow cytometry

Supplementary Information

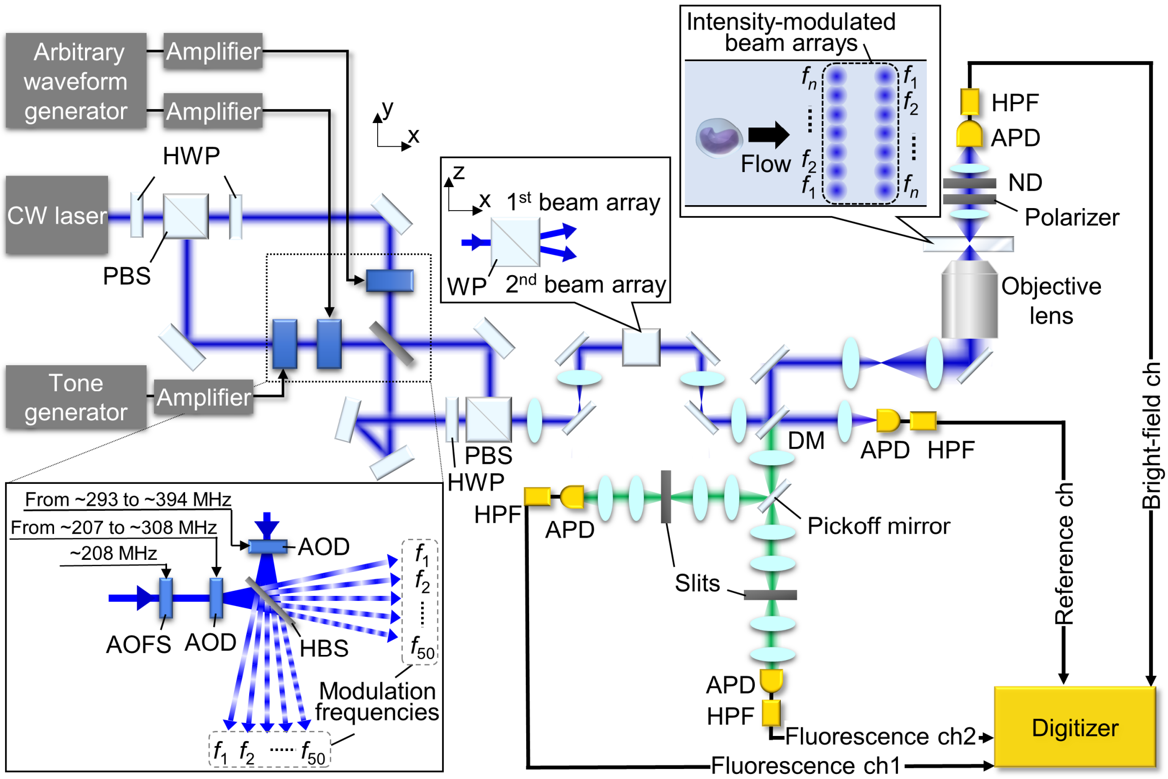

Supplementary Fig. 1 | Detailed schematic of the FLIM flow cytometer. CW: continuous wave; PBS: polarizing beam splitter; HWP: half-wave plate; APD: avalanche photodetector; HPF: high-pass filter; DM: dichroic mirror; HBS: half beam splitter; AOD: acousto-optic deflector; AOFS: acousto-optic frequency shifter; WP: Wollaston prism; ND: neutral density filter; ch: channel. f_1: 20.9960937 MHz, f_2: 25.0976562 MHz, f_{50}: 221.972656 MHz.

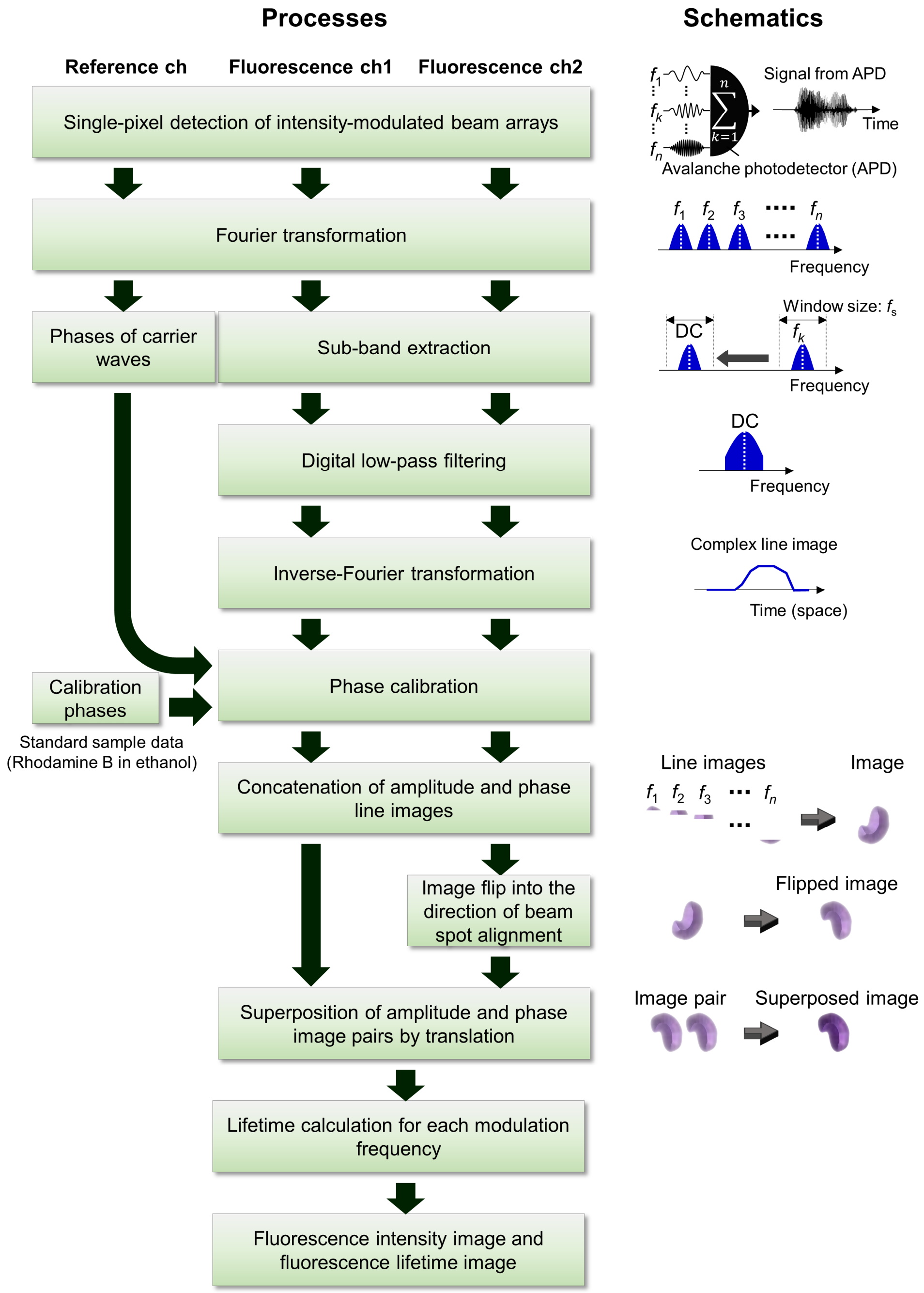

Supplementary Fig. 2 | Signal processing procedure for image reconstruction in the FLIM flow cytometer. Each mount-shaped spectrum indicates the Fourier-transformed sample profile in a line that is scanned by each beam spot. This signal processing was implemented by a lab-made LabVIEW program. The calibration phases were obtained by measuring 10-μM Rhodamine B in ethanol. ch: channel. DC represents a direct-current position (i.e., the zero-frequency position). Modulation frequency: f_k, $1 \leq k \leq n$. f_s represents the modulation frequency spacing between neighboring beam spots (4.1015625 MHz).

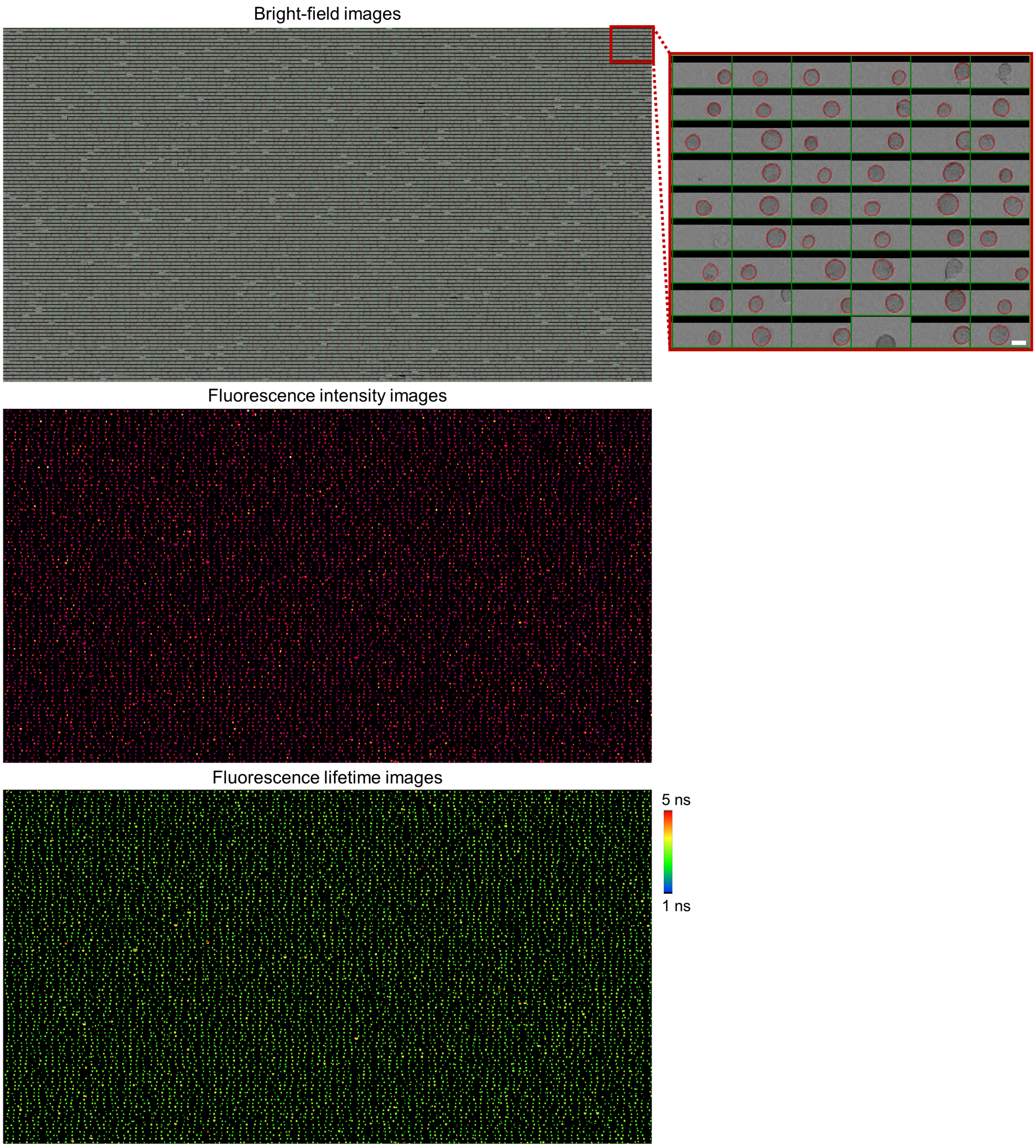

Supplementary Fig. 3 | Example images (100 × 100) obtained by FLIM flow cytometry for event rate calculation. All the images of Jurkat cells stained with Calcein-AM were obtained within 0.83 seconds. In the bright-field images, the cells used for event rate calculation are circled in red. Scale bar: 10 µm. Image acquisitions were repeated eight times, resulting in similar results.

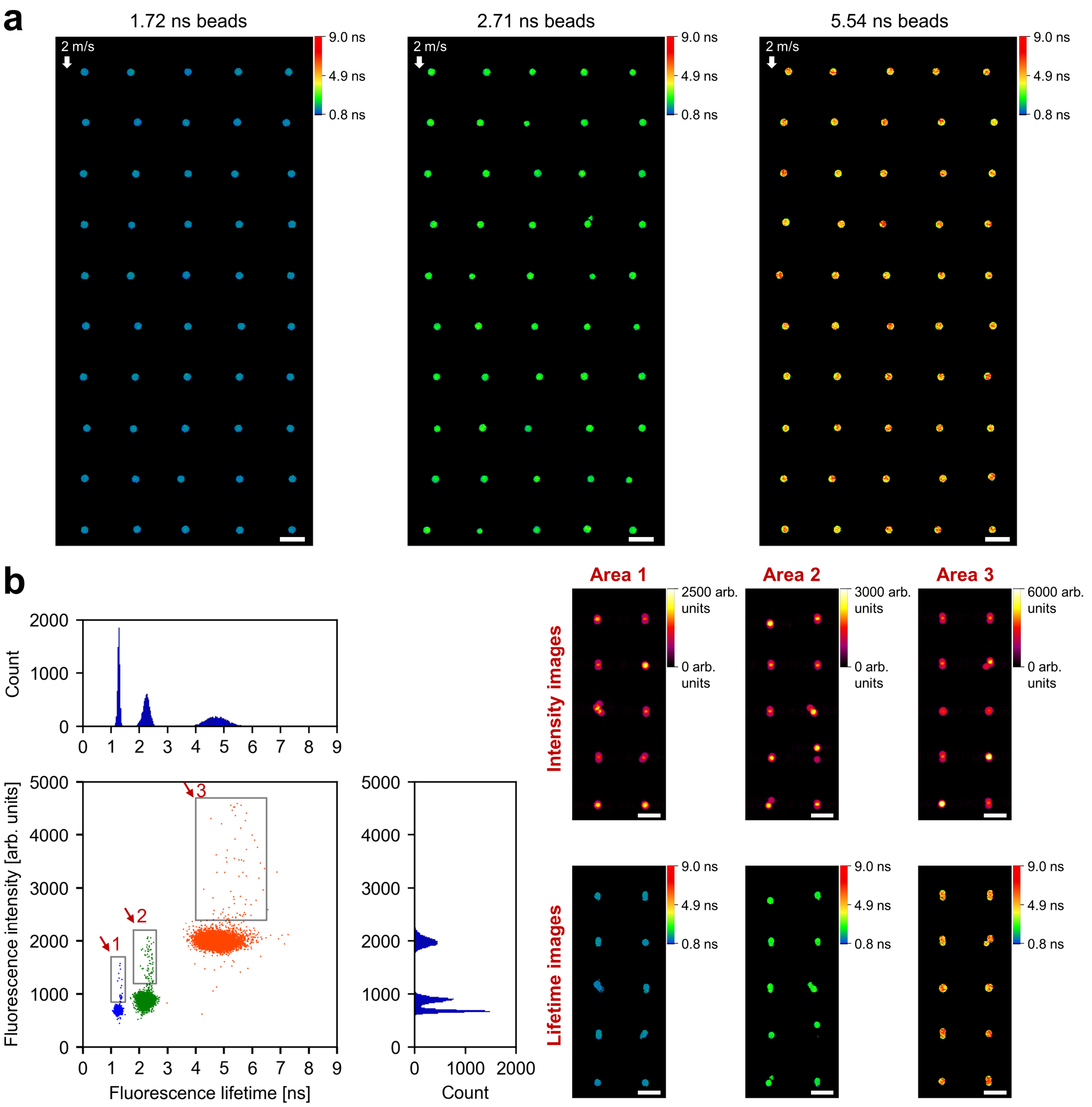

Supplementary Fig. 4 | Separate measurements of different types of beads with FLIM flow cytometry. The same polymer beads as those used in Fig. 3 were measured separately. Scale bars: 20 µm. **a**, Representative fluorescence lifetime images of 6.5-µm polymer beads with fluorescence lifetime values of 1.72 ns, 2.71 ns, and 5.54 ns. The flow speed was 2 m/s. Image acquisitions were repeated more than ten times, resulting in similar results. **b**, Distributions of the fluorescent beads (blue: 1.72 ns beads, n = 5,905; green: 2.71 ns beads, n = 5,930; orange: 5.54 ns bead,: n = 5,984). The calculated fluorescence lifetime values for each type of beads were 1.27 ± 0.04 ns, 2.24 ± 0.13 ns, and 4.75 ± 0.41 ns (mean ± standard deviation), consistent with the results (Fig. 3). The right images show the beads randomly picked from the corresponding gray boxes in the scatter plot. Source data are provided as a Source Data file.

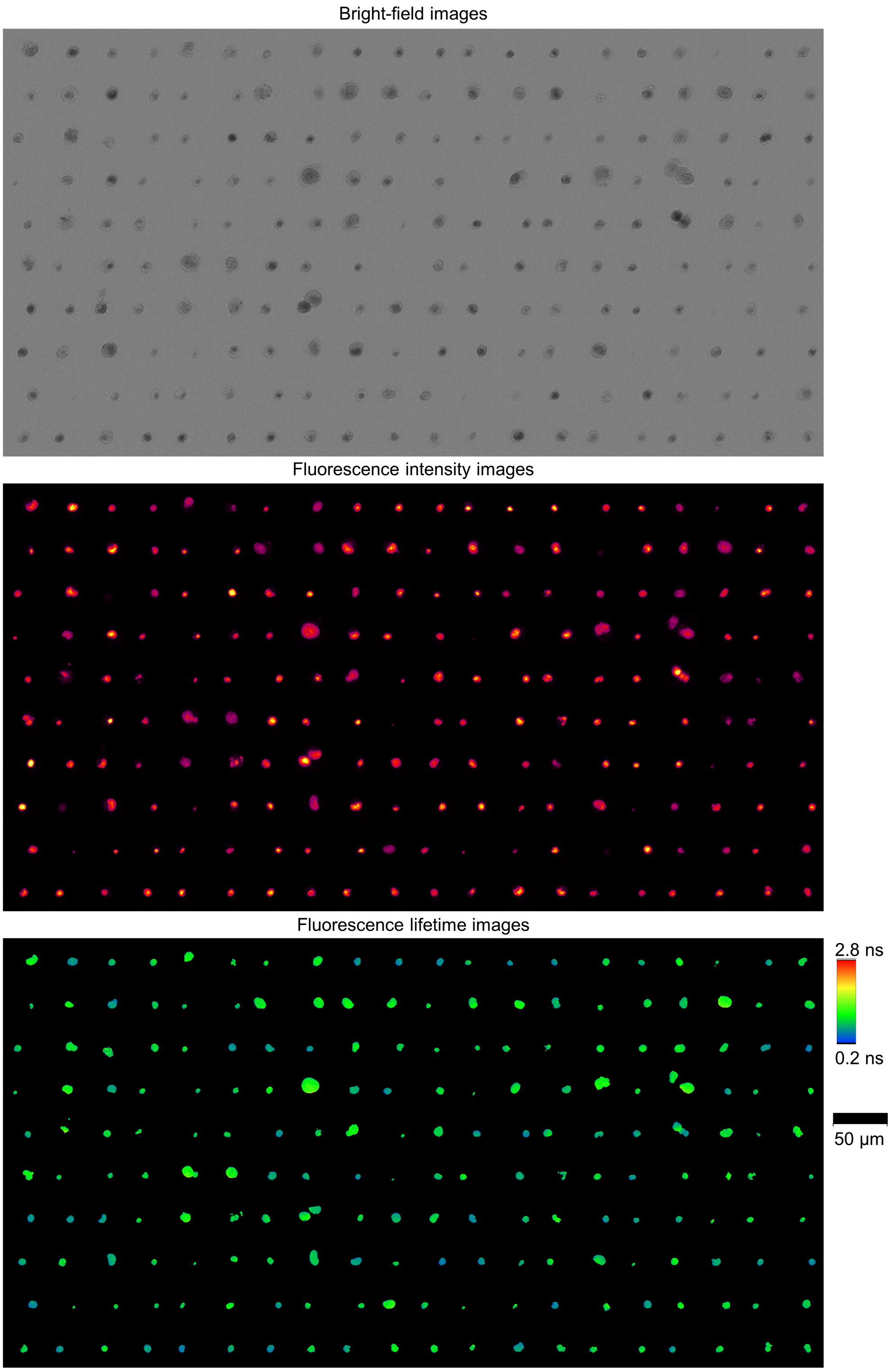

Supplementary Fig. 5 | Representative images (10 × 20) of the tumor-derived cells (Fig. 5) obtained by FLIM flow cytometry. Image acquisitions were repeated more than ten times, resulting in similar results.

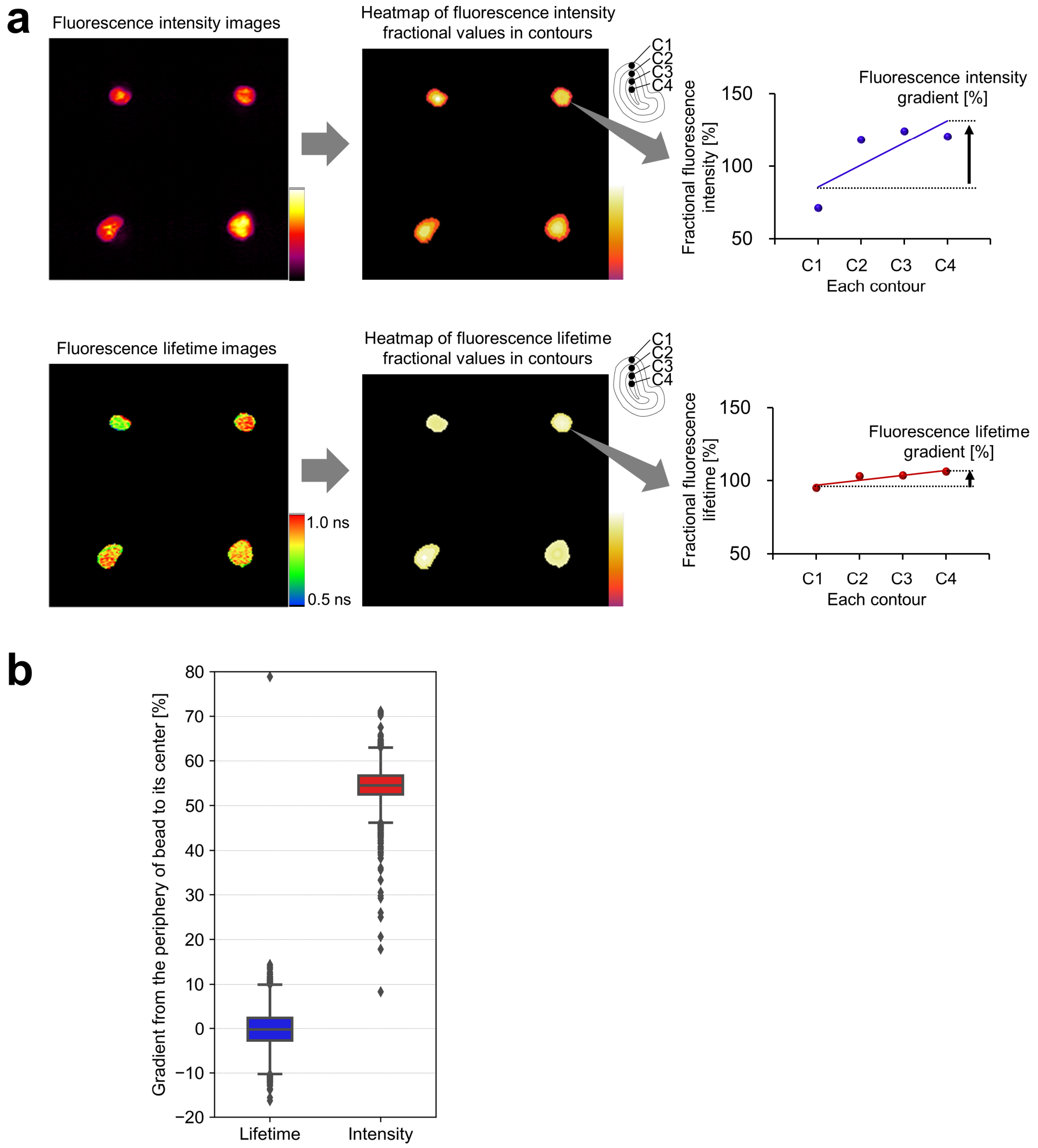

Supplementary Fig. 6 | Fluorescence intensity and fluorescence lifetime gradients from the periphery of an object to the center. a, Schematic illustrating the calculation method. The fluorescence intensity and lifetime gradients indicate the degree of their differences from the outer area (C1) to the inner area (C4) compared to the average **b**, Distributions of fluorescence lifetime and fluorescence intensity gradients calculated using the beads data from Fig. 2, which serve as control data to exclude the possibility of non-biological factors contributing to the fluorescence lifetime gradient. Lifetime: n = 9,496, median = -0.25%, standard deviation (SD) = 4.07%; Intensity: n = 9,496, median = 54.5%, SD = 3.50%. The box plots display the median values with lines inside boxes, the first and third quartiles with box edges, 1.5 times the interquartile range (IQR) with whiskers, and outliers with dots. Source data are provided as a Source Data file.

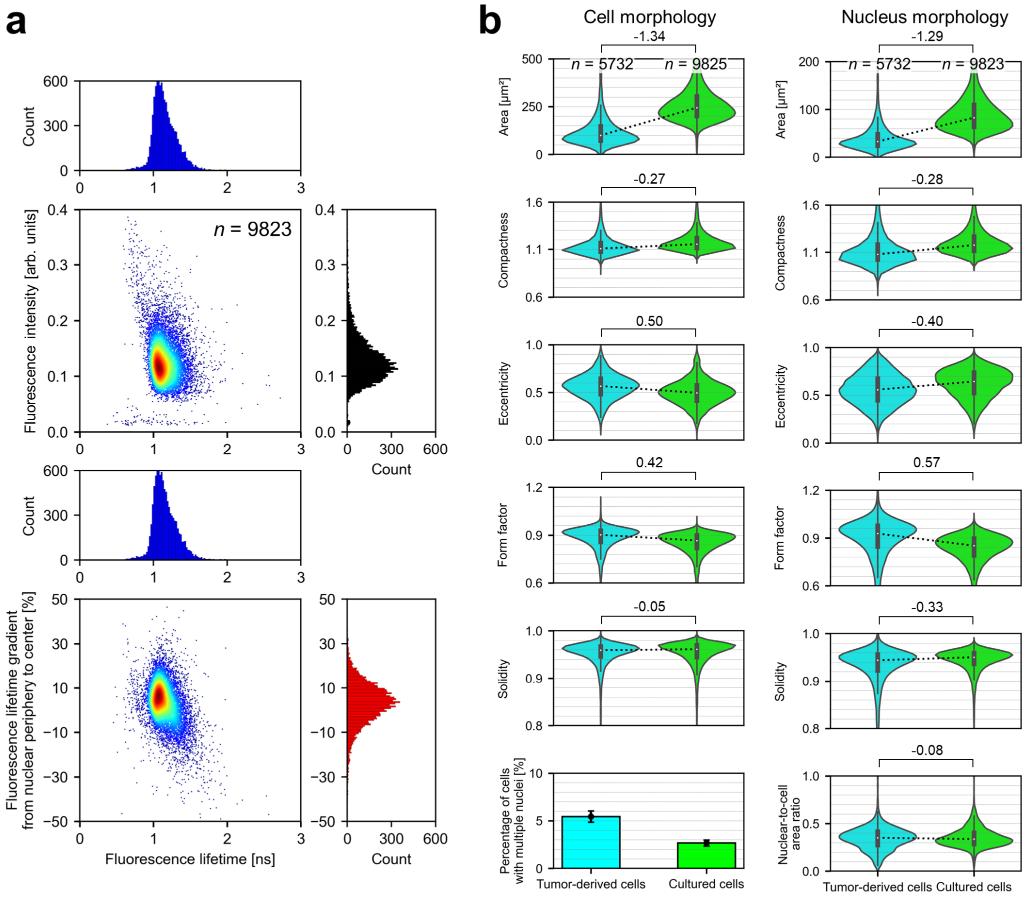

Supplementary Fig. 7 | FLIM flow cytometry of cultured GS-9L cells. a, Distribution of cultured rat glioma cells (GS-9L) stained with SYTO16 in fluorescence intensity (black histogram), fluorescence lifetime (blue histogram), and fluorescence lifetime gradient from nuclear periphery to center (red histogram). n = 9,823. **b**, Comparison of tumor-derived rat glioma cells and cultured rat glioma cells (GS-9L) in cell and nucleus morphological features. The bar plot illustrates the percentage of cells with multiple nuclei calculated for each cell population, accompanied by the upper and lower bounds of its 95% confidence interval. Values between adjacent two samples represent effect sizes (Cohen's d), with their standard errors less than 0.02. The violin plots display the median values with white dots, the first and third quartiles with box edges, and 1.5 times the IQR with whiskers. Source data are provided as a Source Data file.

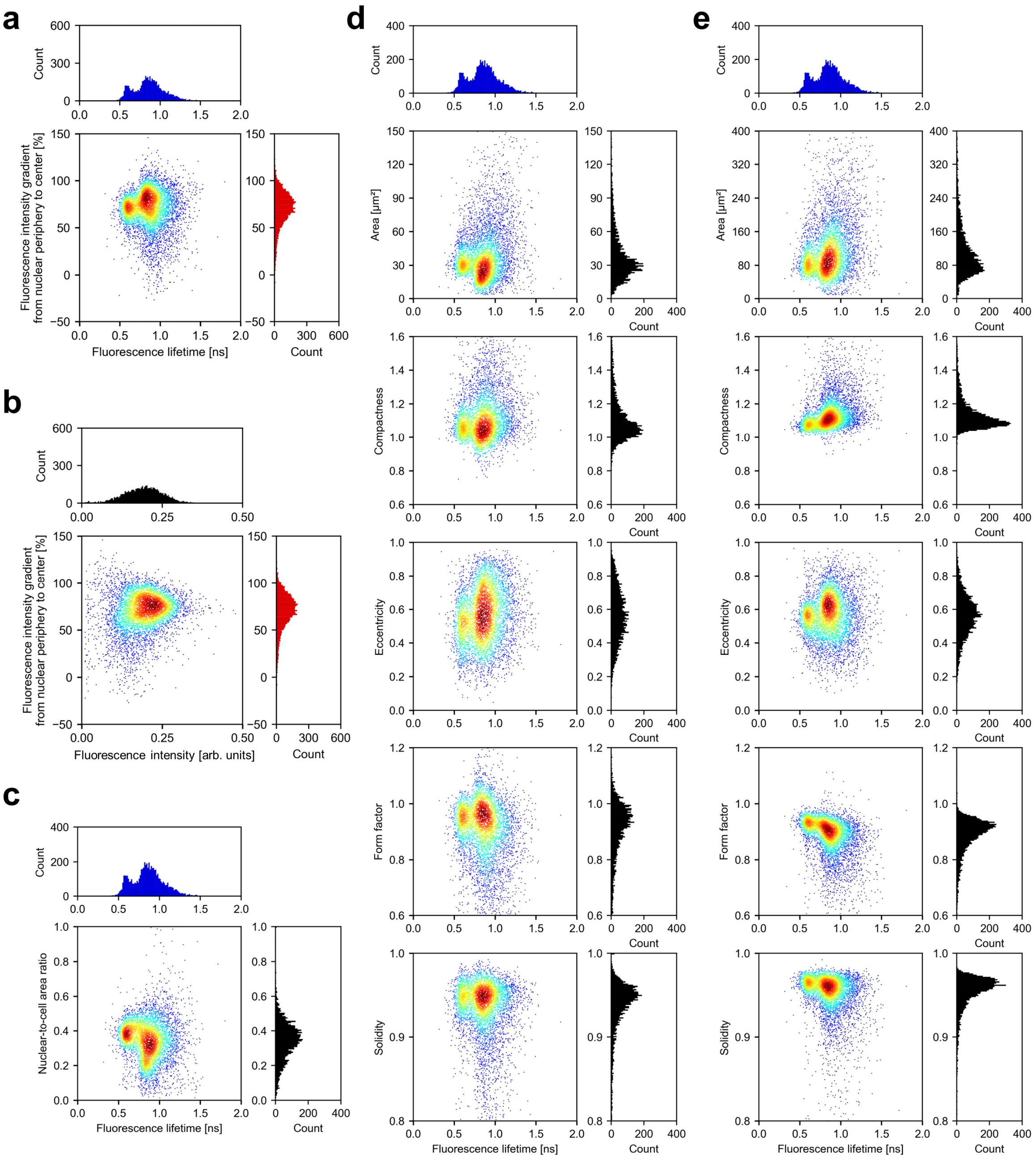

Supplementary Fig. 8 | Distribution of tumor-derived cells (*n* = 5,732) in combinations of morphological features, fluorescence intensity values, and fluorescence lifetime. a, Distribution of the cells in fluorescence lifetime and fluorescence intensity gradient. **b,** Distribution of the cells in fluorescence intensity and fluorescence intensity gradient. **c,** Distribution of the cells in fluorescence lifetime and nuclear-to-cell area ratio. **d,** Distribution of the cells in fluorescence lifetime and several morphological features of their nuclei. **e,** Distribution of the cells in fluorescence lifetime and several morphological features of the entire cells. Source data are provided as a Source Data file.

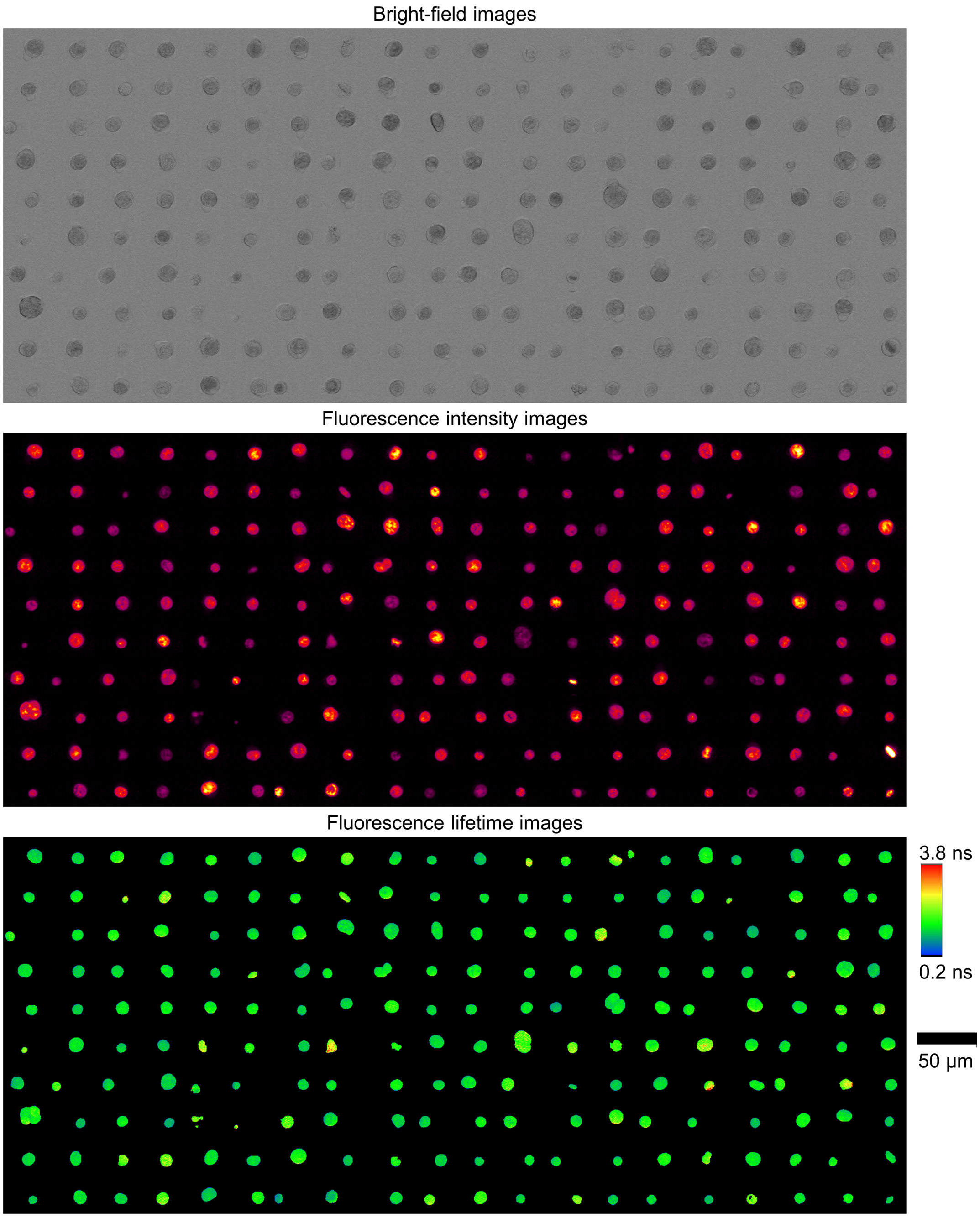

Supplementary Fig. 9 | Representative images (10 × 20) of the control cells stained with SYTO16 (Fig. 6), obtained by FLIM flow cytometry. Image acquisitions were repeated more than ten times, resulting in similar results.

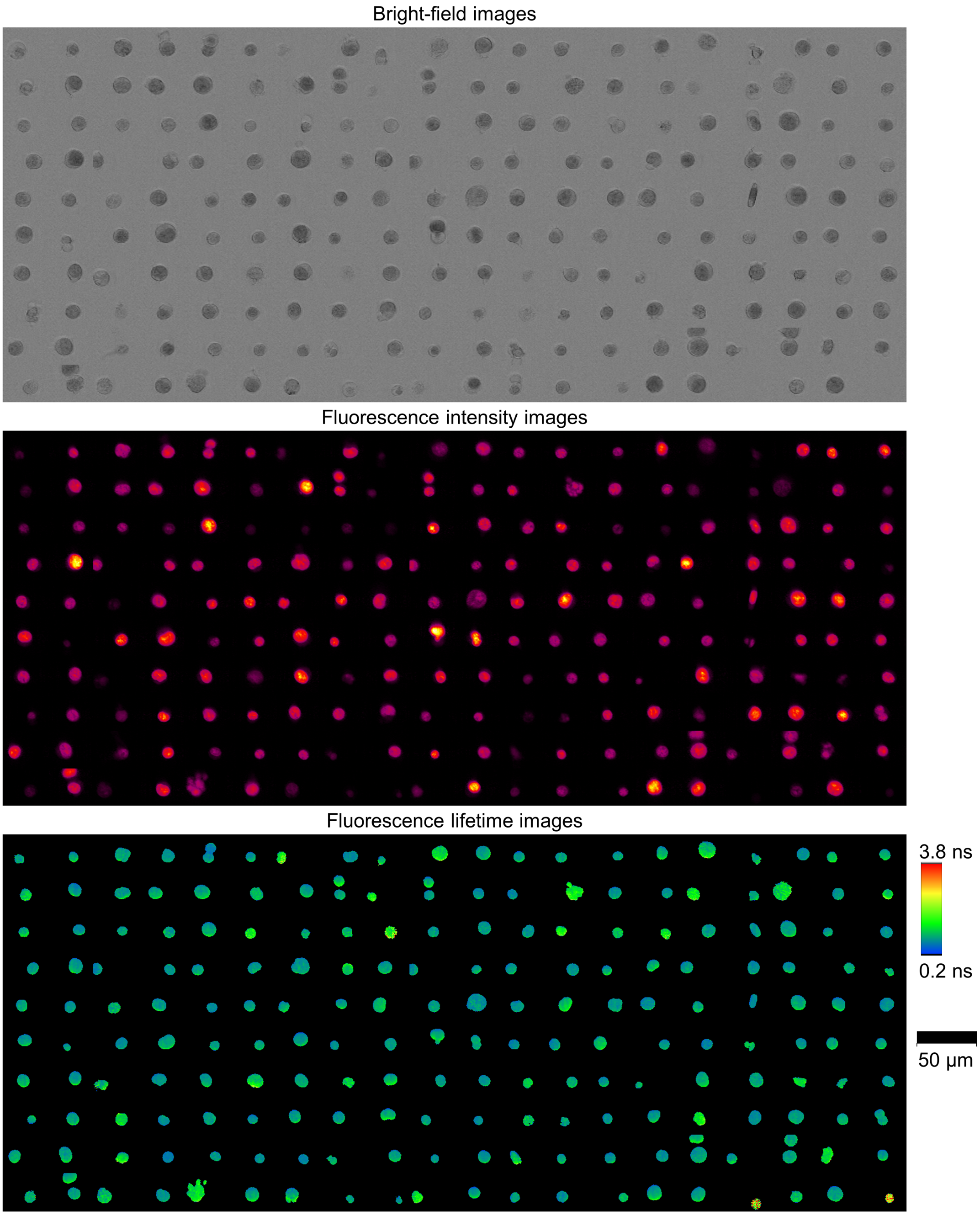

Supplementary Fig. 10 | Representative images (10 × 20) of the Jurkat cells, treated with doxorubicin for 110 min and stained with SYTO16 (Fig. 6), obtained by FLIM flow cytometry. Image acquisitions were repeated more than ten times, resulting in similar results.

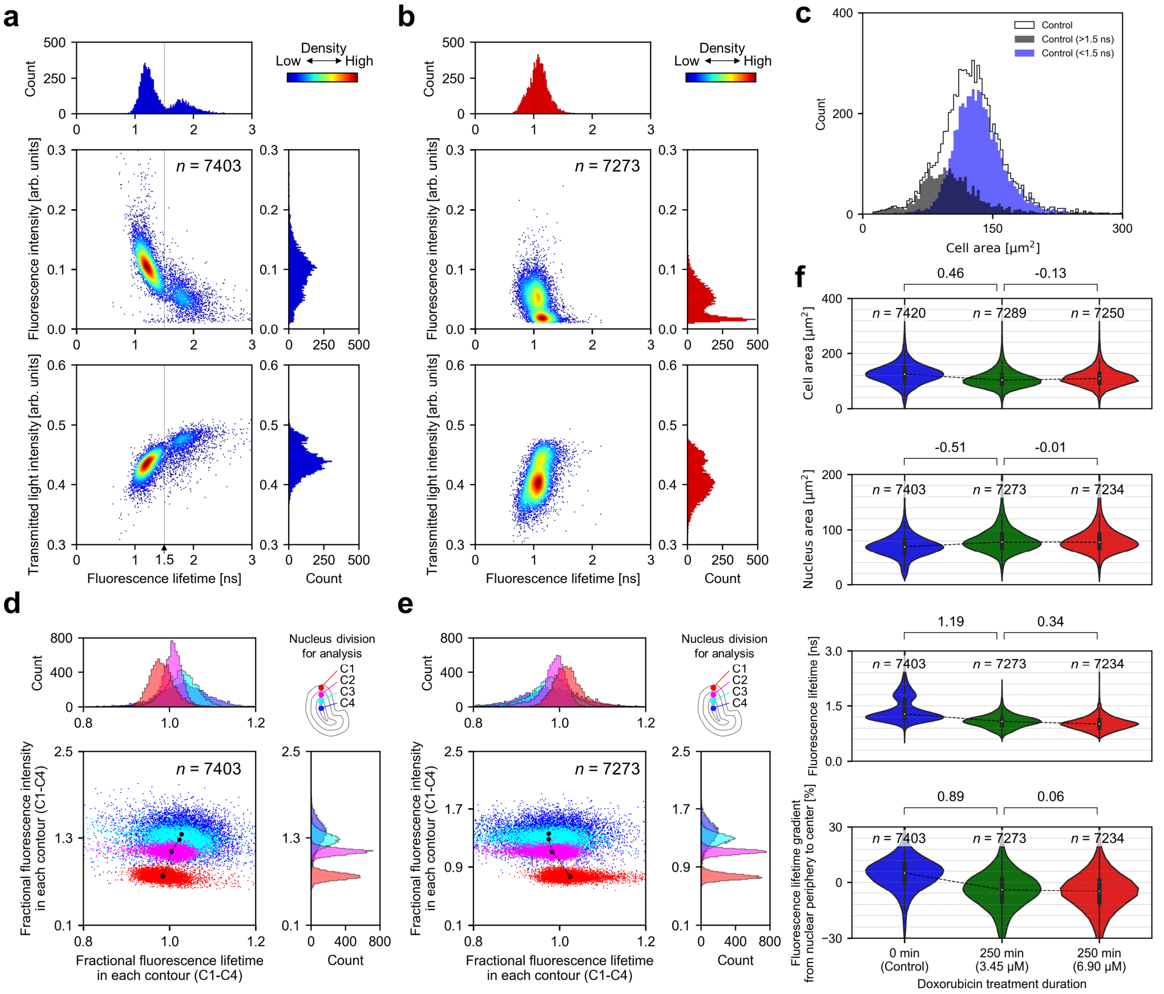

Supplementary Fig. 11 | FLIM flow cytometry of Jurkat cells before and after doxorubicin treatment for 250 min. a, b, Distributions of control (**a**) and 3.45-μM 250-min doxorubicin-treated Jurkat cells (**b**) in fluorescence lifetime, fluorescence intensity, and transmitted light intensity. **c,** Distribution of control cells in cell area. The cells were further separated into two sub-populations with a cut-off fluorescence lifetime value of 1.5 ns (*n* = 2,142 for >1.5 ns, *n* = 5,261 for <1.5 ns). **d, e,** Distributions of control (**d**) and 3.45-μM 250-min doxorubicin-treated Jurkat cells (**e**) in fractional fluorescence lifetime and fractional fluorescence intensity in each contour, which were calculated as the mean values in each contour (C1: outer, C2: middle outer, C3: middle inner, C4: inner contours) divided by the mean values in the entire nucleus. The black dot indicates the average for each area. **f,** Comparison of control cells, as well as those treated with 3.45-μM and 6.90-μM doxorubicin for 250 min in terms of cell area, nucleus area, fluorescence lifetime, and fluorescence lifetime gradient from nuclear periphery to center. Values between every two adjacent samples represent effect sizes (Cohen's *d*), with their standard errors less than 0.02. The violin plots display the median values with white dots, the first and third quartiles with box edges, and 1.5 times the IQR with whiskers. Source data are provided as a Source Data file.

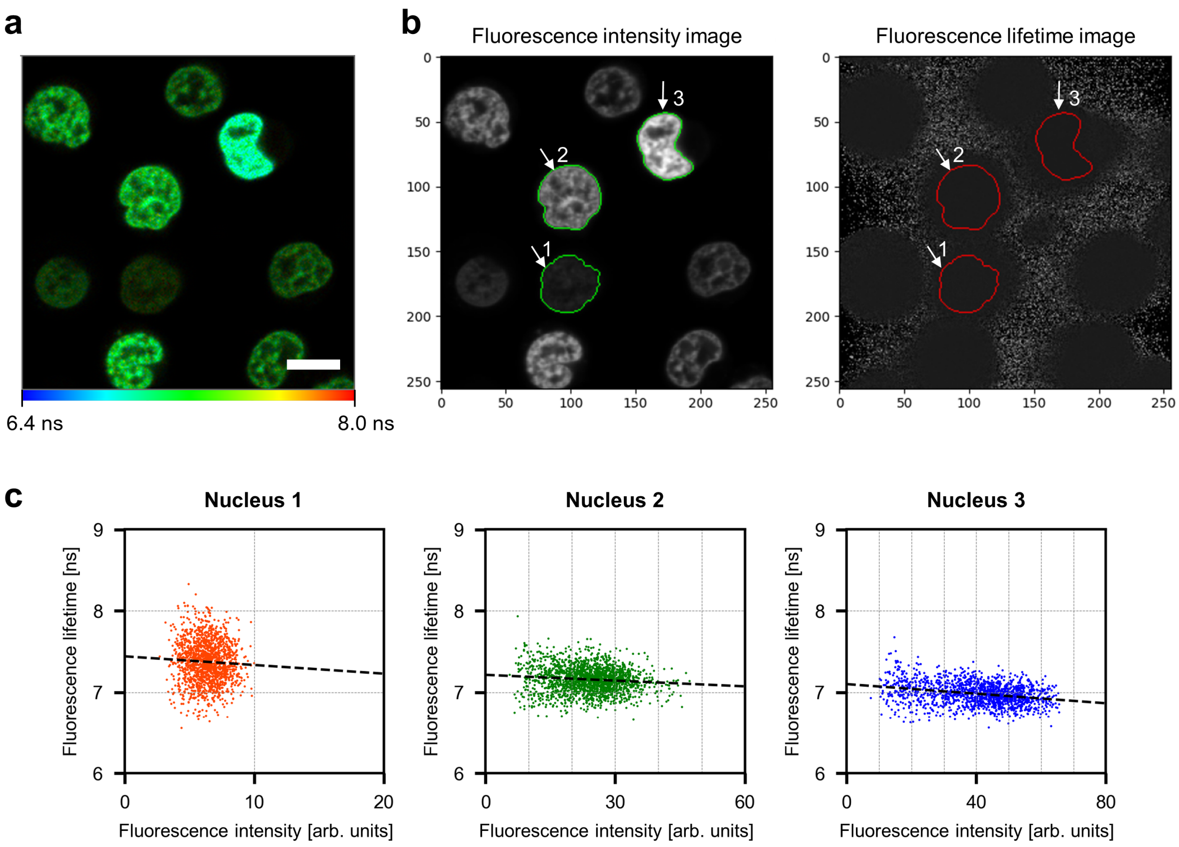

Supplementary Fig. 12 | Analysis of Jurkat cells imaged with confocal TCSPC-based FLIM. a, Representative fluorescence lifetime image of Jurkat cells. Scale bar: 10 µm. Image acquisitions were repeated more than two times, resulting in similar results. **b**, Gray-scale fluorescence intensity and fluorescence lifetime images corresponding to panel **a**. **c**, Scatter plots showing pixel distributions in fluorescence intensity and fluorescence lifetime corresponding to the segmented nuclei indicated by the arrows in panel **b**. The black dashed lines indicate linear fits based on the least square method. Nucleus 1 (n = 1,583); Nucleus 2 (n = 1,919); Nucleus 3 (n = 1,531). Source data are provided as a Source Data file.

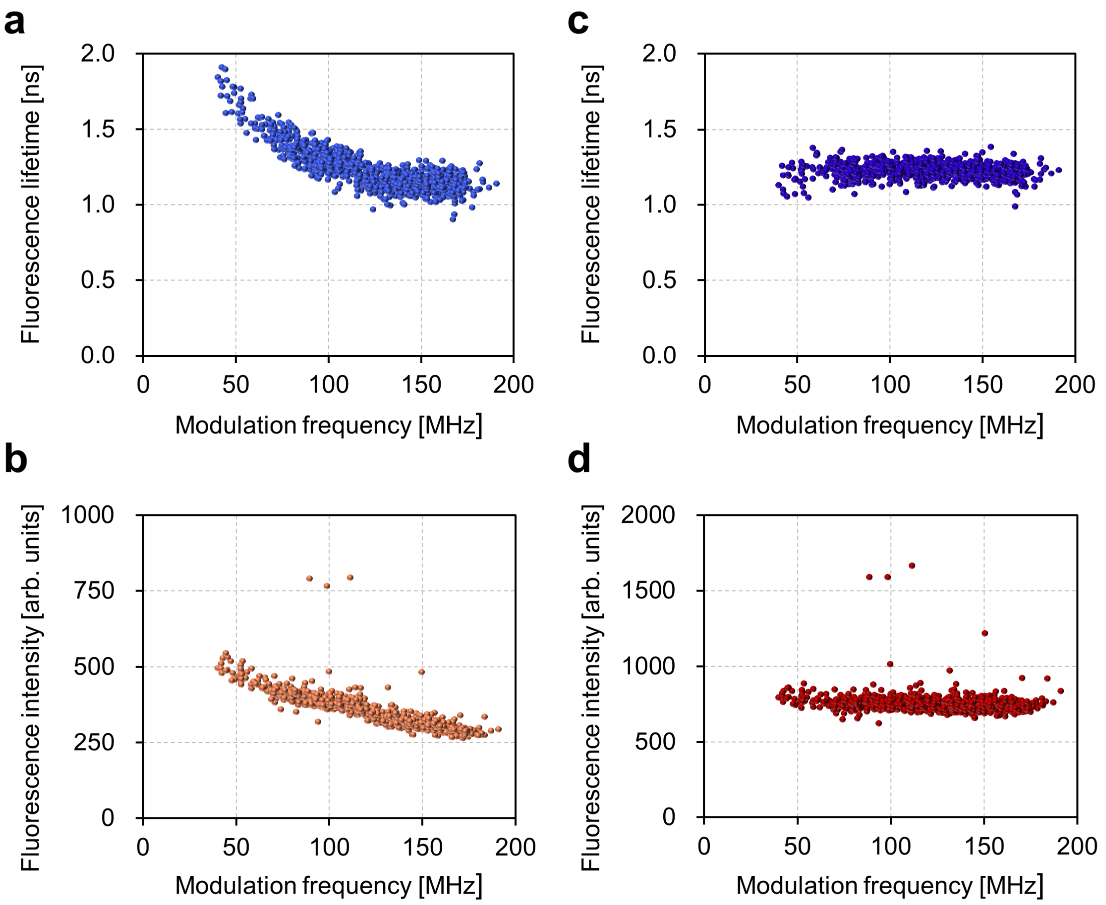

Supplementary Fig. 13 | Effectiveness of dual intensity-modulated beam arrays in enhancing the precision of the FLIM flow cytometer. Fluorescence intensity and lifetime of 1.72 ns polymer beads (PolyAn 11000006) flowing at 3.2 m/s were measured using a single beam array only (**a**, **b**) or dual beam arrays (**c**, **d**). The modulation-frequency-dependent artifacts observed in graph **a** can be attributed to the non-mono exponential fluorescence decay of the beads, whereas those in graph **b** primarily stemmed from the fluorescence lifetime of the beads, regardless of it being non mono-exponential decay. The modulation frequency was calculated as the mean of the modulation frequencies from the single beam array used for fluorescence lifetime imaging of each bead. **a,** Relationship between modulation frequencies and fluorescence lifetimes. The standard deviation (SD) of fluorescence lifetimes was 0.144 ns. **b**, Relationship between modulation frequencies and fluorescence intensities. The coefficient of variation (CV) of fluorescence intensity was 0.153. **c,** Relationship between modulation frequencies and fluorescence lifetimes. The SD of fluorescence lifetimes was 0.048 ns. **d**, Relationship between modulation frequencies and fluorescence intensities. The CV of fluorescence intensity was 0.080. The raw data for the beads (n = 1,000) were the same in panels **a**-**d**. Source data are provided as a Source Data file.

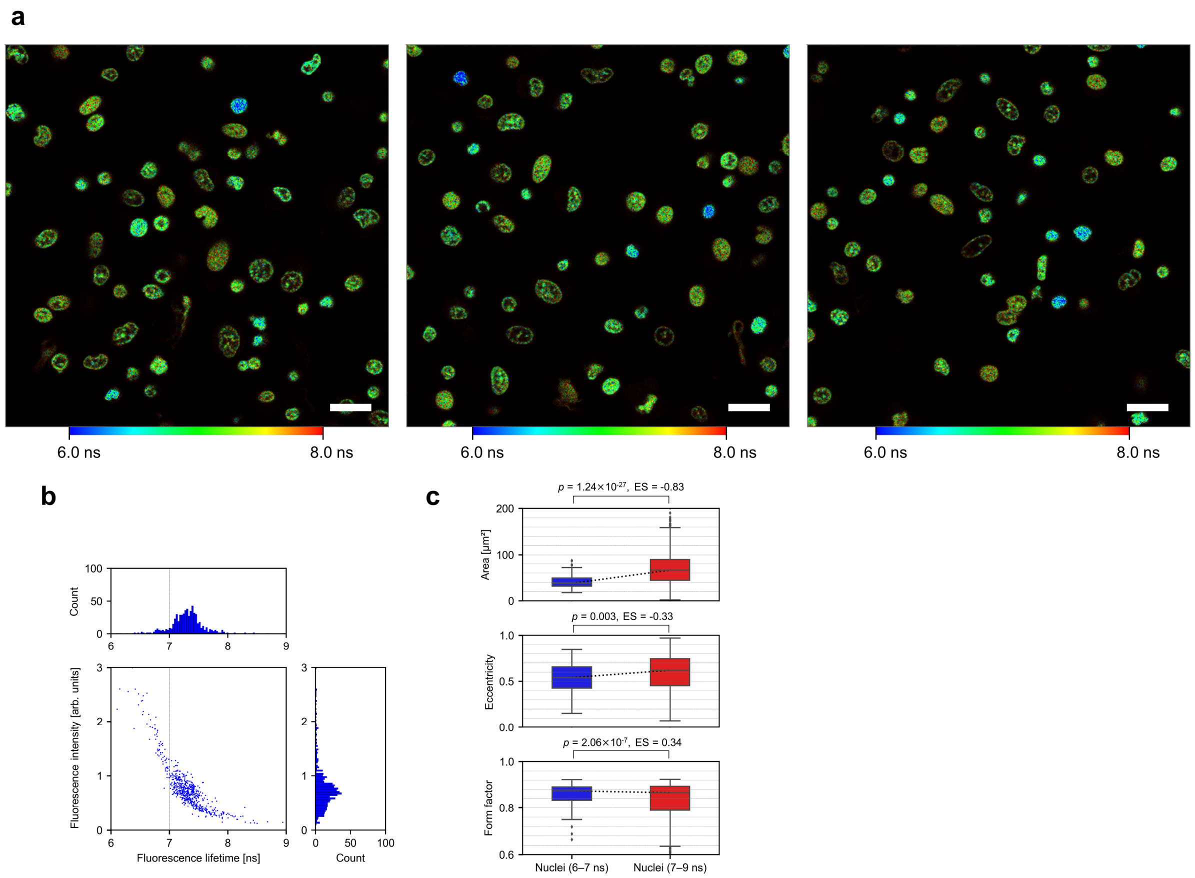

Supplementary Fig. 14 | Analysis of tumor-derived rat glioma cells imaged with TCSPC-based confocal FLIM. a, Representative fluorescence lifetime images of the cells derived from a male rat glioma obtained with confocal TCSPC-based FLIM. Scale bars: 20 μm. Image acquisitions were repeated more than ten times, resulting in similar results. **b**, Scatter plot of the cells in terms of fluorescence lifetime and fluorescence intensity. n = 640. **c**, Comparison of morphological features for the nuclei with shorter (<7 ns) and longer (>7 ns) fluorescence lifetimes. The box plots display the median values with lines inside boxes, the first and third quartiles with box edges, 1.5 times the IQR with whiskers, and outliers with dots. ES represents effect size (Cohen's d). p values were calculated with Welch's t-test (two-sided). Note that differences between segmentation algorithms used in FLIM flow cytometry and those used in image analysis for confocal microscopy may result in variations in the absolute values of nuclear sizes obtained by both methods. Source data are provided as a Source Data file.
