# Supplementary material for: High-throughput fluorescence lifetime imaging flow cytometry
Source: Nat Commun. 2024 Sep 4;15:7376. doi: 10.1038/s41467-024-51125-y (PMC11375057; doi:10.1038/s41467-024-51125-y)
Supplement: Supplementary file 2 — Reporting Summary [file 41467_2024_51125_MOESM2_ESM.pdf]

nature portfolio

Corresponding author(s): Hiroshi Kanno, Keisuke Goda

Last updated by author(s): Jul 30, 2024

Reporting Summary

Nature Portfolio wishes to improve the reproducibility of the work that we publish. This form provides structure for consistency and transparency in reporting. For further information on Nature Portfolio policies, see our Editorial Policies and the Editorial Policy Checklist.

Statistics

For all statistical analyses, confirm that the following items are present in the figure legend, table legend, main text, or Methods section.

n/a	Confirmed	
☐	☒	The exact sample size (*n*) for each experimental group/condition, given as a discrete number and unit of measurement
☐	☒	A statement on whether measurements were taken from distinct samples or whether the same sample was measured repeatedly
☐	☒	The statistical test(s) used AND whether they are one- or two-sided<br>*Only common tests should be described solely by name; describe more complex techniques in the Methods section.*
☒	☐	A description of all covariates tested
☒	☐	A description of any assumptions or corrections, such as tests of normality and adjustment for multiple comparisons
☐	☒	A full description of the statistical parameters including central tendency (e.g. means) or other basic estimates (e.g. regression coefficient) AND variation (e.g. standard deviation) or associated estimates of uncertainty (e.g. confidence intervals)
☐	☒	For null hypothesis testing, the test statistic (e.g. *F*, *t*, *r*) with confidence intervals, effect sizes, degrees of freedom and *P* value noted<br>*Give P values as exact values whenever suitable.*
☒	☐	For Bayesian analysis, information on the choice of priors and Markov chain Monte Carlo settings
☒	☐	For hierarchical and complex designs, identification of the appropriate level for tests and full reporting of outcomes
☐	☒	Estimates of effect sizes (e.g. Cohen's *d*, Pearson's *r*), indicating how they were calculated

Our web collection on statistics for biologists contains articles on many of the points above.

Software and code

Policy information about availability of computer code

Data collection	LabVIEW2016 and LabVIEW2021 were used for data collection. The programs used for data collection in this study cannot be made publicly available because they include vendor-provided functions for specific devices, such as an arbitrary waveform generator and a digitizer.
Data analysis	CellProfiler 4.2.1 was used for image analysis for FLIM flow cytometry. In addition, LabVIEW2021 and Python 3.10.9 were used for data analysis. To analyze images obtained with TCSPC-based confocal FLIM, Python 3.10.7 and OpenCV 4.5.1.48 library were used. All the codes used for data analysis in this study are available in the Figshare database [https://doi.org/10.6084/m9.figshare.26403916].

For manuscripts utilizing custom algorithms or software that are central to the research but not yet described in published literature, software must be made available to editors and reviewers. We strongly encourage code deposition in a community repository (e.g. GitHub). See the Nature Portfolio guidelines for submitting code & software for further information.

Data

Policy information about availability of data

All manuscripts must include a data availability statement. This statement should provide the following information, where applicable:

- Accession codes, unique identifiers, or web links for publicly available datasets
- A description of any restrictions on data availability
- For clinical datasets or third party data, please ensure that the statement adheres to our policy

The image and other associated raw data generated in this study have been deposited in the Figshare database [https://doi.org/10.6084/m9.figshare.26403916] and [https://doi.org/10.6084/m9.figshare.26403919]. Source data are provided with this paper.

Research involving human participants, their data, or biological material

Policy information about studies with human participants or human data. See also policy information about sex, gender (identity/presentation), and sexual orientation and race, ethnicity and racism.

Reporting on sex and gender	n/a
Reporting on race, ethnicity, or other socially relevant groupings	n/a
Population characteristics	n/a
Recruitment	n/a
Ethics oversight	n/a

Note that full information on the approval of the study protocol must also be provided in the manuscript.

Field-specific reporting

Please select the one below that is the best fit for your research. If you are not sure, read the appropriate sections before making your selection.

☒ Life sciences ☐ Behavioural & social sciences ☐ Ecological, evolutionary & environmental sciences

For a reference copy of the document with all sections, see nature.com/documents/nr-reporting-summary-flat.pdf

Life sciences study design

All studies must disclose on these points even when the disclosure is negative.

Sample size	The number of images used in this study per sample typically ranged 5,000 to 10,000 to ensure statistical significance comparable to the standard practice in flow cytometry analyses (see each figure for the precise sample size for each experiment). No statistical methods were used to predetermine the sample sizes.
Data exclusions	Objects that were not identified by CellProfiler in the images were excluded from the analysis. In addition, several objects that returned NaN (Not a Number) values for their morphological features were also excluded. Moreover, a small number of outliers were not shown in the figures to ensure better visibility of the data distributions. Instead, all data points are provided in the Source Data.
Replication	The experimental findings were validated through multiple independent experiments. There were rat glioma samples that did not fully replicate the same cell distributions, potentially due to variations in in vivo tumor conditions (e.g., differences in cell composition ratios).
Randomization	To avoid potential systematic errors during data collection by the FLIM flow cytometer, the measurement order of the multiple samples was randomized where applicable (e.g., Fig. 6). The measurement order is provided in the manuscript.
Blinding	The investigators were not blinded to allocation during experiments and outcome assessment because a single investigator conducted both the measurements and the analysis.

Reporting for specific materials, systems and methods

We require information from authors about some types of materials, experimental systems and methods used in many studies. Here, indicate whether each material, system or method listed is relevant to your study. If you are not sure if a list item applies to your research, read the appropriate section before selecting a response.

Materials & experimental systems

n/a	Involved in the study	
x		Antibodies
	x	Eukaryotic cell lines
x		Palaeontology and archaeology
	x	Animals and other organisms
x		Clinical data
x		Dual use research of concern
x		Plants

Methods

n/a	Involved in the study	
x		ChIP-seq
	x	Flow cytometry
x		MRI-based neuroimaging

Eukaryotic cell lines

Policy information about cell lines and Sex and Gender in Research

Cell line source(s)	Both Jurkat (E6.1) and GS-9L cells used in this study were derived from ECACC.<br>Jurkat cell were purchased through KAC (Cat. No. EC88042803-F0).
Authentication	Jurkat cell lines were authenticated by ECACC (STR profiling). GS-9L was not authenticated.
Mycoplasma contamination	Both cell lines were negative for mycoplasma contamination, certified by ECACC, but that was not tested after using them.
Commonly misidentified lines<br>(See ICLAC register)	No commonly misidentified cell lines were used in this study.

Animals and other research organisms

Policy information about studies involving animals; ARRIVE guidelines recommended for reporting animal research, and Sex and Gender in Research

Laboratory animals	Rat, Fischer 344 (F344/NSlc), Male, 10 weeks old.
Wild animals	n/a
Reporting on sex	Only male rats were used in this study. Sex differences were not considered in this study, as its aim was the proof-of-principle demonstration of the developed FLIM flow cytometer.
Field-collected samples	n/a
Ethics oversight	All animal experiments were approved by the Animal Studies Ethics Committee of Tohoku University Graduate School of Medicine and were conducted at a certified laboratory in Tohoku University Graduate School of Medicine (2021MdA-087-02). Humane endpoints were defined as when rats exhibited symptoms such as decreased activity, reduced water intake, decreased food intake, rapid weight loss (more than 20% in a few days), obvious signs of pain, or prominent indicators of central nervous system failure. We strictly adhered to these guidelines.

Note that full information on the approval of the study protocol must also be provided in the manuscript.

Plants

Seed stocks	n/a
Novel plant genotypes	n/a
Authentication	n/a

Flow Cytometry

Plots

Confirm that:

☐ The axis labels state the marker and fluorochrome used (e.g. CD4-FITC).

☐ The axis scales are clearly visible. Include numbers along axes only for bottom left plot of group (a 'group' is an analysis of identical markers).

☐ All plots are contour plots with outliers or pseudocolor plots.

☒ A numerical value for number of cells or percentage (with statistics) is provided.

Methodology

Sample preparation	All biological samples were stained with SYTO16 (Thermo Fisher Scientific, Cat. No. S7578) or Calcein-AM (Funakoshi, Cat. No. 341-07901) in an incubator (37°C, 5% CO2) for approximately 60 minutes at the concentrations of 1–5 µM. After washing off the staining solution, the cells were suspended in PBS and then subjected to FLIM flow cytometry measurement. See the Methods section for detailed staining conditions for each experiment. All non-biological samples (i.e., beads) were measured by FLIM flow cytometry in their original form. In Fig. 3, the beads solution were combined in equal volumes.
Instrument	Harvard Apparatus, H70-4500
Software	The custom code used in this study have been deposited in the Figshare database [https://doi.org/10.6084/m9.figshare.26403916].
Cell population abundance	No cell sorting was performed in this study.
Gating strategy	Event acquisitions were triggered by detecting fluorescence signal emitted from flowing objects. These events were converted to images. Objects that were not identified by CellProfiler in the images or that returned NaN (Not a Number) values for their morphological features were excluded from analysis to prevent the inclusion of noise. In Fig. 2, the criteria for objects were an eccentricity of less than 0.6 for beads and an eccentricity of less than 0.72 for cells (Supplementary Fig. 3). In other experiments, such criteria were not used. Cell separation in Fig. 5b was performed manually.

☐ Tick this box to confirm that a figure exemplifying the gating strategy is provided in the Supplementary Information.
